# Assessment of free-hand transperineal targeted prostate biopsy using multiparametric magnetic resonance imaging-transrectal ultrasound fusion in Chinese men with prior negative biopsy and elevated prostate-specific antigen

**DOI:** 10.1186/s12894-017-0241-3

**Published:** 2017-07-05

**Authors:** Huibo Lian, Junlong Zhuang, Wei Wang, Bing Zhang, Jiong Shi, Danyan Li, Yao Fu, Xuping Jiang, Weimin Zhou, Hongqian Guo

**Affiliations:** 1 Department of Urology, Drum Tower Hospital, Medical School of Nanjing University, 321 Zhongshan Road, Nanjing, 210008 Jiangsu People’s Republic of China; 2 Department of Radiology, Drum Tower Hospital, Medical School of Nanjing University, 321 Zhongshan Road, Nanjing, 210008 Jiangsu People’s Republic of China; 3 Department of Pathology, Drum Tower Hospital, Medical School of Nanjing University, 321 Zhongshan Road, Nanjing, 210008 Jiangsu People’s Republic of China; 4 Institute of Urology, Nanjing University, Nanjing, 210008 Jiangsu People’s Republic of China; 5 Department of Urology, the Affiliated Yixing people’s Hospital of Jiangsu University, Yixing, Jiangsu 212000 China

**Keywords:** Magnetic resonance imaging, Prostate cancer, Repeat biopsy, Targeted biopsy, Transrectal ultrasound

## Abstract

**Background:**

To evaluate the role of free-hand transperineal targeted prostate biopsy using multiparametric magnetic resonance imaging-transrectal ultrasound (mpMRI-TRUS) fusion in Chinese men with repeated biopsy.

**Methods:**

A total of 101 consecutive patients suspicious of prostate cancer (PCa) at the mpMRI scan and with prior negative biopsy and elevated PSA values were prospectively recruited at two urological centers. Suspicious areas on mpMRI were defined and graded using PI-RADS score. Targeted biopsies (TB) were performed for each suspicious lesion and followed a 12-core systematic biopsy (SB). Results of biopsy pathology and whole-gland pathology at prostatectomy were analyzed and compared between TB and SB. The risk for biopsy positivity was assessed by univariate and multivariate logistic regression analysis.

**Results:**

Fusion biopsy revealed PCa in 41 of 101 men (40.6%) and 25 (24.8%) were clinically significant. There was exact agreement between TB and SB in 74 (73.3%) men. TB diagnosed 36% more significant cancer than SB (22 vs 13 cases, *P* = 0.012). When TB were combined with SB, an additional 14 cases (34.1%) of mostly significant PCa (71.4%) were diagnosed (*P* = 0.036). TB had greater sensitivity and accuracy for significant cancer than SB in 26 men with whole-gland pathology after prostatectomy. PI-RADS score on mpMRI was the most powerful predictor of PCa and significant cancer.

**Conclusions:**

Free-hand transperineal TB guided with MRI-TRUS fusion imaging improves detection of clinical significant PCa in Chinese men with previously negative biopsy. PI-RADS score is a reliable predictor of PCa and significant cancer.

**Electronic supplementary material:**

The online version of this article (doi:10.1186/s12894-017-0241-3) contains supplementary material, which is available to authorized users.

## Background

Since the 1980s, transrectal ultrasound (TRUS) guided systematic prostate biopsy is performed on patients with abnormal serum prostate-specific antigen (PSA) or suspicious digital rectal examination [1]. This conventional method has been shown to have limited sensitivity for detecting prostate cancer (PCa), which of the false-negative rate may be as high as 47% [2]. PSA related anxiety and repeated biopsy dilemma consist in many of the men with negative biopsies and persistently elevated serum PSA levels [3]. Approximately 38% of them undergo a repeat biopsy within 5 years with cancer detection only in an additional 13 to 41% [4].

In order to improve biopsy sensitivity, the concept of targeted biopsy (TB) on suspicious areas through magnetic resonance imaging (MRI) guidance was established [5]. Although multiparametric MRI (mpMRI) offered an increased sensitivity and specificity on prostate biopsy guidance, the disadvantage of time-consuming and equipment-specialization made it not widely used [6]. Recently, MRI-TRUS fusion technique has been developed and proposed, because of its combination of the soft tissue resolution of MRI and the practicability of TRUS [7]. The mpMRI-TRUS image fusion biopsy system is a novel fusion technology which not only provides visualization of both recorded multiplanar reconstruction images on that one monitor, but also real-timely makes diagnostic or procedural decisions [8]. Using one such fusion device, we got initial encouraging result for targeted prostate biopsy as reported by other researchers [9–11]. Unfortunately, targeted MRI-TRUS fusion biopsy has not been well evaluated with free-hand transperineal approach, especially in Asian men with previously negative biopsy.

We present double center results to evaluate the impact of using mpMRI-TRUS image fusion technology for free-hand transperineal TB in Chinese men with prior negative biopsy and elevated PSA, and compare biopsy performance between TB and 12-core systematic biopsy (SB) in the cancer detection.

## Methods

### Study population

After the approval of institutional review board, a prospective study of free-hand transperineal TB guided with MRI-TRUS fusion imaging was performed at two Chinese urological centers from May 2014 to March 2016 (Drum Tower Hospital, Medical School of Nanjing University, and the Affiliated Yixing people’s Hospital of Jiangsu University). One hundred and one consecutive patients with at least one prior negative prostate biopsy and persistently elevated serum PSA levels were included. All of them were evaluated with prostate mpMRI and considered having at least one suspicious area in mpMRI images.

### Multiparametric MRI

All mpMRI was performed using a 3 Tesla MRI scanner (Achieva; Philips Medical System, The Netherlands) with a 32-channel phased array coil. The protocol of acquisition of different MRI sequences was recently published [12]. Images analyses were performed and supervised by two experienced uroradiologists. Suspicious areas were defined and a likelihood score from 2 to 5 for each lesion was provided according to the Prostate Imaging Reporting and Data System (RI-PADS) [13] based on the European Society of Urogenital Radiology prostate MRI guidelines [14]. We didn’t use the PI-PADS Version 2 [15], because the new system had not been well validated and most of the patients’ lesions were evaluated before its publication.

### Biopsy procedure

**Fig. 1:**
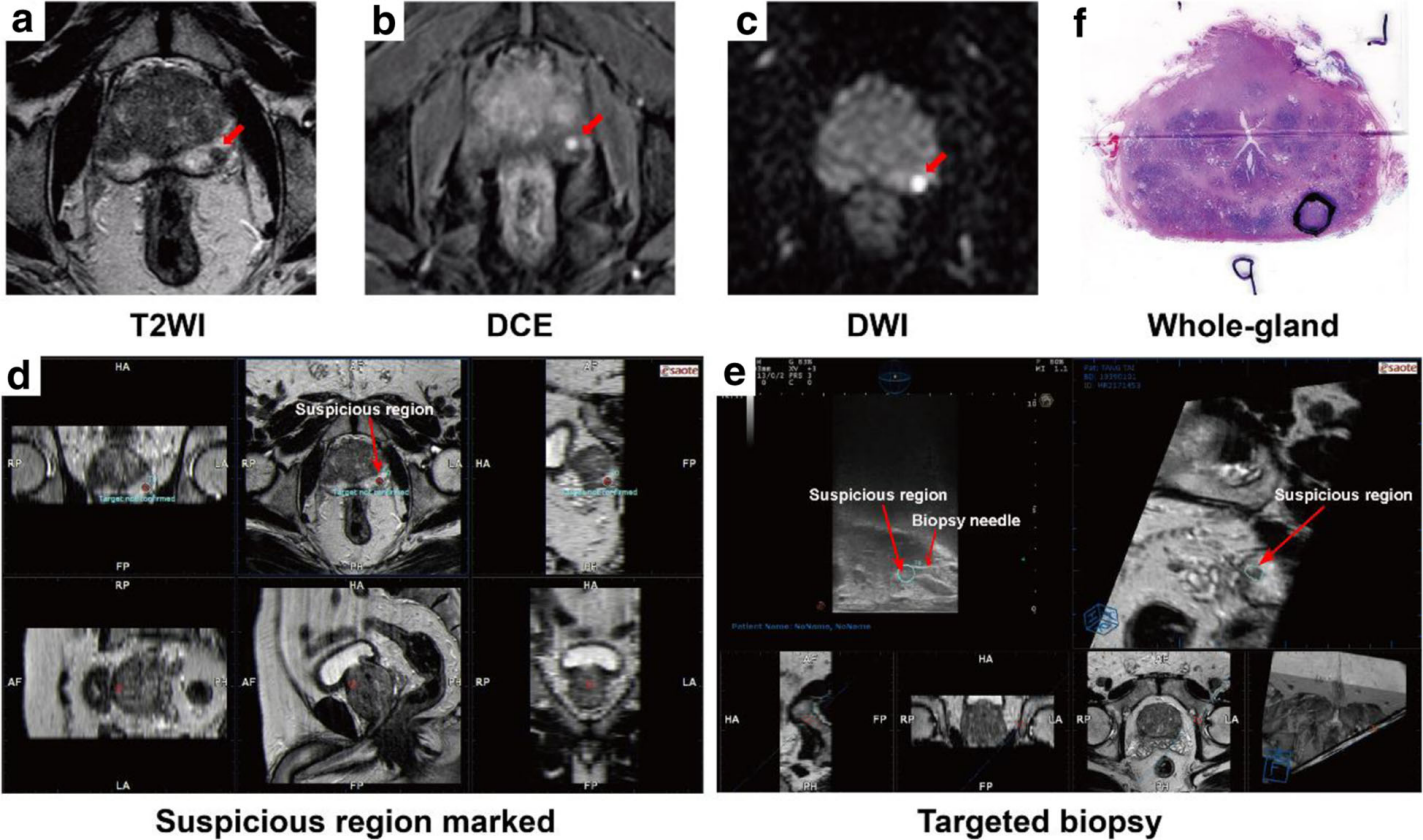
Steps for mpMRI and TRUS fusion guided targeted prostate biopsy and whole-gland prostatectomy pathology in a typical patient with negative biopsy two years ago but elevated prostate-specific antigen. A-C, The lesion (*red arrow*) was detected in different mpMRI sequences and was scored as “probably malignant” (score 4 of 5). D-E, mpMRI data was loaded into the biopsy system and suspicious region (*greeb circle*) was marked on high resolution transversal T2 W sequences before biopsy. Then, the targeted biopsy started using the free-hand transperineal technique guided by mpMRI and TRUS fusion images (*red arrow*). F, Targeted cores revealed Gleason 3 + 4 tumor in the lesion (90% core involvement). Whole-gland pathology was performed after prostatectomy and the index lesion (*black circle*) had the same pathology result with targeted biopsy

All biopsies were performed with an mpMRI-TRUS fusion guided biopsy technique (RVS®, Real-time Virtual Sonography, Hitachi Medical Corporation, Tokyo, Japan) and 18-G automatic biopsy guns with 22 mm specimen size (Bard Magnum; Bard Medical, Covington, GA, USA) as described previously [12]. In brief, morphological MRI data was loaded into the biopsy system and suspicious areas were marked on high resolution transversal T2 W sequences (Fig. 1 a-d). Then, ultrasound probe with magnetic position sensor was used to get the TRUS images. During fusing two kinds of images, the MR images reconstructed from the MRI volume data were corresponded to the ultrasound sagittal images using internal urethral orifice as the fiducial landmark. Thus, mpMRI data with the marked suspicious lesions were real-timely superimposed on the TRUS images at the same monitor.

Then, the biopsy started with TB using the free-hand transperineal technique without the guide of template by one experienced urologist. Cancer-suspicious lesions identified on MRI were semiautomatically displayed on the real-time TRUS image to guide biopsy needle [10]. During free-hand transperineal biopsy procedure, the puncture point was chose to keep away from pubis and adjacent organs, and make the needle correspond to the ultrasound sagittal images (Fig. 1 e). Every targeted lesion was biopsied at least each one core in axial and sagittal planes. Standard 12-core SB using same transperineal approach was carried out afterwards by another experienced urologist who was blinded to the MRI targeted lesions. During the biopsy procedure, all patients with lithotomy position underwent general anesthesia using a larynx mask.

### Pathological analysis

All biopsies and prostatectomy whole mount pathology were examined and analyzed by two senior pathologists (J.S. and Y.F.). The highest Gleason score from the TB or the standard 12-core SB was determined for each patient. Clinically significant cancer on biopsy was defined as Gleason score 3 + 4 or higher or Gleason score 6 with maximal cancer core length ≥ 4 mm [16, 17]. This definition was selected in an effort to incorporate both grade and volume, and avoid the bias caused by multiple cores from the same tumor.

Each prostatectomy specimen was processed using the modified Stanford technique, with 5 mm transverse step-sectioned samples taken from the apex to the base and the sagittal section of the distal 5–8 mm of the apex and base [18]. The step sectioned specimens were denoted as the apex, middle, or base of the prostate for analyses of three equal trisections of the prostate. Pathologists were blinded to the MRI and TRUS imaging results. The index tumor lesion in prostatectomy specimens was defined as the lesion with extraprostatic extension, the highest Gleason score, or the largest volume if Gleason scores were the same, in order of priority. The pathology slide with the greatest cross-section of the index lesion was used for location matching analysis. The tumor center of the index lesion was defined as the point of intersection of the lesion height and width dimensions and was registered retrospectively in the 27-ROI schema by urologists [19]. Significant PCa at prostatectomy histology was defined using active surveillance criteria (total tumor volume ≥ 0.7 ml or Gleason score > 3 + 4) [20]. The Steps for mpMRI and TRUS fusion guided targeted prostate biopsy and whole-gland prostatectomy pathology are outlined in Fig. 1.

### Data statistics

Statistical analyses were performed using SPSS version 17.0 (SPSS, Inc., Chicago, IL). The Fisher’s exact test was used to compare categorical variables. Univariate analysis was applied with one-way ANOVA test. Multivariate logistic regression analysis was performed to identify potential predicted factors for the positive result of biopsy. Data was presented as mean ± SD. A *P* value <0.05 was considered statistically significant.

## Results

Patient demographics and summary of fusion-guided biopsy findings are shown in Table 1. A total of 101 patients with prior negative biopsy and elevated PSA were suspected to have PCa with a PI-RADS score between 2 and 5 according to mpMRI examination. The mean age of the patient population was 68.9 years (SD 8.1) and mean number of MRI lesions was 1.9 (SD 1.0). The mean pre-fusion-guided biopsy PSA level was 10.8 ng/ml (SD 6.1) and prostate volume was 42.1 ml (SD 15.3). The mean number of targeted biopsy cores per patient was 4.2 (SD 1.5). Of 101 suspected patients, 41 (40.6%) were diagnosed PCa, including 16 (15.8%) insignificant and 25 (24.8%) significant cancers. Twenty-six patients who ultimately underwent prostatectomy were analyzed as a subgroup. Compared with all biopsy populaiton, patients who underwent prostatectomy were younger (65.2 vs 68.9 years, *P* = 0.028), had smaller prostate volumes (35.1 vs 42.1 ml, *P* = 0.015), had more MRI lesions (2.4 vs 1.9, *P* = 0.029), and had more TB cores (4.9 vs 4.2, *P* = 0.040).

**Table 1:** Patient demographics and summary of fusion-guided biopsy findings

	All patients	Men with PCa	Prostatectomy cohort
Men, no.	101	41 (40.6)	26 (25.7)
Age, year	68.9 ± 8.1	67.8 ± 8.0	65.2 ± 7.2
PSA, ng/ml	10.8 ± 6.1	11.3 ± 6.3	11.2 ± 6.3
Prostate volume, ml	42.1 ± 15.3	39.4 ± 13.6	35.1 ± 11.8
Prior negative biopsy, no.	1.5 ± 0.7	1.4 ± 0.6	1.4 ± 0.6
MRI lesions per patient, no.	1.9 ± 1.0	2.1 ± 1.1	2.4 ± 1.0
PI-RAD score, no. (%)			
2	13 (12.9)	1 (1.0)	0 (0)
3	31 (30.7)	3 (3.0)	0 (0)
4	36 (35.6)	19 (18.8)	12 (11.9)
5	21 (20.8)	18 (17.8)	14 (13.9)
TB cores per patiens	4.2 ± 1.5	4.8 ± 1.6	4.9 ± 1.8
Insignificant PCa	-	16 (15.8)	6 (5.9)
Significant PCa	-	25 (24.8)	20 (19.8)
Gleason score, no. (%)			
Gleason 6	-	17 (16.8)	6 (5.9)
Gleason 7 (3 + 4)	-	9 (8.9)	8 (7.9)
Gleason 7 (4 + 3)	-	7 (6.9)	5 (5.0)
Gleason ≥8	-	8 (7.9)	7 (6.9)

Continuous variables reported as mean ± standard deviation

*PSA* prostate-specific antigen, *MRI* magnetic resonance imaging, *PCa* prostate cancer, *SB* systematic biopsy, *TB* targeted biopsy

The comparative pathologic outcomes of prostate systemic biopsy and targeted biopsy are shown in Table 2 and Additional file 1: Table S1. Seventy-four patients (60 + 4 + 10) of the total population (73.3%) demonstrated exact agreement between TB and SB. TB diagnosed a similar PCa number (31 cases) to SB (27 cases). However, TB diagnosed 36% more significant cancers than SB (22 vs 13 cases, *P* = 0.012). Among the 16 cases (4 + 10 + 2, 15.8%) in which TB revealed a higher risk category from SB group, 12 (10 + 2, 75%) were upgraded to significant cancers; whereas in 11 cases (8 + 2 + 1, 10.9%) which SB demonstrated a higher risk category from TB group, only 3 (2 + 1, 27.3%) were upgraded to significant cancers (*P* = 0.022). In addition, the utility of TB alone lead to 10 less cases of cancer (24.4%), only 2 (20%) of these were significant. However, SB alone missed 14 cases of cancer (34.1%) and 10 (71.4%) were significant (*P* = 0.036). In other words, when TB were combined with SB, an additional 14 cases of mostly significant PCa (10 cases) were diagnosed.

**Table 2:** Comparison of pathology from systematic biopsy and targeted biopsy for prostate cancer

	SB			
	No cancer	Insignificant cancer	Significant cancer	Totals
TB				
No cancer.	60	8	2	70
Insignificant cancer	4	4	1	9
Significant cancer	10	2	10	22
Totals	74	14	13	101

*SB* systematic biopsy, *TB* targeted biopsy

The subgroup of 26 patients who underwent prostatectomy was also analyzed because pathology results from TB and SB could be compared against the whole-gland prostatectomy pathology (Table 3 and Additional file 1: Table S1). Within this subcohort, nine patients (the sum of all “no cancer” values for SB, 1 + 1 + 1 + 6) were diagnosed with PCa preoperatively only by TB, of whom 6 (66.7%) were significant cancer on whole-gland pathology. By contrast, 4 patients were diagnosed with PCa only by SB, only 1 (25%) were significant cancer on whole-gland pathology. When assessing the ability of preoperative biopsy to predict whole-gland pathology significance, the sensitivity of TB were 85% versus 45% for SB (*P* = 0.019), while the specificities were same (83.3%). The total accuracy of TB were 84.6% versus 53.8% for SB (*P* = 0.034).

**Table 3:** Comparison of whole-mount prostatectomy outcome with target biopsy and systematic biopsy pathology for prostate cancer

	Whole-Mount Pathology (Prostatectomy)				
	Insig cancer		Sig cancer		Totals
TB	SB		SB		
No cancer	No cancer	0	No cancer	0	4
	Insig cancer	2	Insig cancer	1	
	Sig cancer	1	Sig cancer	0	
Insig cancer	No cancer	1	No cancer	1	4
	Insig cancer	1	Insig cancer	1	
	Sig cancer	0	Sig cancer	0	
Sig cancer	No cancer	1	No cancer	6	18
	Insig cancer	0	Insig cancer	2	
	Sig cancer	0	Sig cancer	9	
Totals	6		20		26

*SB* systematic biopsy, *TB* targeted biopsy, *Insig* Insignificant, *Sig* significant

In order to identify any potential predictor associated with detection of PCa and significant cancer, univariate and multivariate analysis were performed (Table 4). PI-RADS score was significantly correlated with both the PCa and significant PCa (both *P* < 0.001). Age, MRI lesions and PSA value of patients was only correlated with PCa (*P* = 0.028, *P* < 0.001 and *P* = 0.03). The further multivariate analysis revealed that PSA value and PI-RADS score were independent predictive factors of the positive biopsy of PCa (*P* = 0.004, OR = 1.22; *P* = 0.001, OR = 3.64). Moreover, patients with high PI-RADS scores (4, 5) had an over 10-fold higher risk of positive biopsy compared to those with low PI-RADS scores (2, 3). Additional file 2: Figure S1 also showed a strong relationship between PI-RADS score and biopsy results.

**Table 4:** Univariate and multivariate analysis (logistic regression) predicting prostate cancer and clinically significant cancer

	Diagnosed with PCa			Sig PCa
	Univariate	Multivariate		Univariate
	*P* value	*P* value	OR (95% CI)	*P* value
Age	0.028	0.847	1.01 (0.93–1.10)	0.812
PSA	<0.001	0.004	1.22 (1.07–1.39)	0.070
Prostate volume	0.114	-	-	0.397
Prior negative biopsy	0.541	-	-	0.483
MRI lesions per patient	0.030	0.193	1.67 (0.77–3.59)	0.953
Biopsy cores	0.165	-	-	0.587
PI-RAD score	<0.001	0.001	3.64 (1.74–7.63)	<0.001
4 + 5 vs 2 + 3	<0.001	<0.001	10.94 (3.0–40.1)	<0.001

*PCa* prostate cancer, *Sig* significant, *PSA* prostate-specific antigen, *MRI* magnetic resonance imaging

## Discussion

Imaging techniques, mainly mpMRI, have developed as an accurate modality in PCa detection. Lesions identified on mpMRI correlate with tumor location on radical prostatectomy specimens [21]. Real-time fusion of mpMRI and TRUS images of the prostate is feasible and potentially able to identify cancerous regions for subsequent biopsy. This kind of biopsy can be performed using MRI localization information without requiring the cost, difficulties, or inconvenience of an MRI suite or MRI-compatible equipment. This double center prospective study evaluated the impact of real time free-hand transperineal targeted prostate biopsy guided by MRI-TRUS fusion imaging and made comparisons of biopsy performance between TB and traditional 12-core SB in Chinese men with prior negative biopsy sessions.

Our study indicated that PCa detection rate of TB and SB was 30.7 and 26.7% respectively, while the overall rate increased to 40.6% when combined the two approaches. With a mean of only 16.2 biopsies, we achieved a comparable overall detection rate to the others. Taira et al. reported a cancer yield ranging from 34.4 to 55.5% for men with 1, 2, and ≥3 prior negative biopsies [2]. They used transperineal template guided mapping biopsy approach with an average of 54 cores. Walz et al. showed a cancer detection rate of 41% by using a 24-core transrectal saturation biopsy in men with at least two prior negative 8-core biopsies [4]. It indicated that MRI-TRUS fusion guided free-hand transperineal biopsy with lower cores obtained higher or almost cancer detection rate compared to transperineal template mapping biopsy or transrectal saturation approach. Our result was similar to Brock’s, with a TB detection rate of 26.7% and overall rate of 40.6% by using transrectal MRI/real-time elastography fusion biopsy [22]. Besides, our overall cancer detection rate seemed higher than Sonn’s result of 34% [23], who used transrectal MRI-TRUS fusion biopsy in men with one or more previously negative biopsies and elevated PSA levels. We considered that the inconsistent result was because of the different biopsy pathway and patient demography, with older mean age and higher average PSA level in our cohort.

Current evidence demonstrates the improved sensitivity for detecting high grade or clinically significant PCa using MRI-TRUS fusion guided TB than with 12-core SB [9, 10, 24]. In this study, TB significantly increased the detection of significant PCa while decreasing the detection of insignificant cancer compared with SB in a repeat biopsy setting. When using the whole-gland pathology significance as the “gold standard”, TB had a greater accuracy than SB for significant cancer on prostatectomy and a higher sensitivity of 85% versus 45%. Thus, our results demonstrated that TB could significantly change the distribution of clinical significance in repeated biopsy patients diagnosed with PCa toward diagnosis of more significant disease.

The European Society of Urogenital Radiology (ESUR) published the PI-RADS to standardize the MRI scoring system in 2012 [14], which had been validated in primary and repeat biopsy cohorts [7, 25]. Portalez, D et al. considered that ESUR scoring system provided a clinically relevant stratification of the risk of showing PCa in the challenging situation of repeat biopsies [25]. Brock M et al. reported that the prediction of PCa and significant cancer was calculated with an AUC of 0.79 and 0.81 for PI-RADS score in lesion of repeat biopsies. Sonn GA et al. showed that image grade [26] of suspicion on MRI was the most powerful predictor of significant cancer on multivariate analysis [23]. In our cohort, using univariate and multivariate analysis, PI-RADS score was proven to be the strongest predictor of PCa or significant cancer as well (Table 4). Moreover, a strong relationship existed between PI-RADS score and biopsy results (Additional file 2: Figure S1). Patients with high PI-RADS scores (4, 5) had an over 10-fold higher risk of biopsy positivity compared to those with low PI-RADS scores (2, 3).

It is well known that the incidence of infectious complications following TRUS guided transrectal prostate biopsy is steadily increased. In a European randomized trial of 10,474 prostate needle biopsies, the febrile complication rate was as high as 4.2% [27]. In the patients of repeated transrectal biopsies, there was a higher chance of acquiring sepsis with organisms resistant to standard antibiotics, such as multiresistant *Escherichia coli* [28]. Recently, there was an increased interest in the use of a transperineal approach for prostate biopsy [28, 29]. Transperineal prostate biopsy has the advantage of avoiding penetration of rectal mucosa and thus minimizing inoculation of the prostate with bowel flora. Many published series of transperineal prostate biopsy reported their incidence of febrile complication with either zero or close to zero [28–30]. In this series, we use the prostate biopsy methodology of free-hand transperineal approach with general anesthesia, and no peri-procedure complication including infectious and anaesthetic complications was noted.

Several limitations of the present study needed to be mentioned. The study population consisted of patients referred to Chinese men in Eastern China, which could have induced selection bias. Second, patients with no lesion on mpMRI were excluded from the study, which could influence cancer detection rate of SB. Third, the sample size was small, which might have an effect on the results of the study.

## Conclusions

This clinical study showed encouraging results for free-hand transperineal targeted prostate biopsy guided with MRI-TRUS fusion imaging in Chinese men with previously negative biopsies and elevated PSA levels. MRI-TRUS fusion guided TB improves detection of clinical significant PCa in a repeat biopsy setting. Combination of TB and SB can maximize the PCa detection rate. PI-RADS score is the strongest predictor of PCa and significant cancer.

## Additional files

Additional file 1: Table S1. Pathology results from systematic biopsy and targeted biopsy for prostate cancer. (DOCX 16 kb)

Additional file 2: Figure S1. Proportion of all cancers and clinically significant cancers stratified by PI-RADS score according to mpMRI scan. PCa, prostate cancer. (DOCX 138 kb)

## Abbreviations

ESUR: European Society of Urogenital Radiology
mp: Multiparametric
MRI: Magnetic resonance imaging
PCa: Prostate cancer
PI-RADS: Prostate Imaging Reporting and Data System
PSA: Prostate-specific antigen
SB: Systematic biopsy
TB: Targeted biopsy
TRUS: Transrectal ultrasound
