# Supplementary material for: Assessment of free-hand transperineal targeted prostate biopsy using multiparametric magnetic resonance imaging-transrectal ultrasound fusion in Chinese men with prior negative biopsy and elevated prostate-specific antigen
Source: BMC Urol. 2017 Jul 5;17:52. doi: 10.1186/s12894-017-0241-3 (PMC5499050; doi:10.1186/s12894-017-0241-3)
Supplement: Supplementary file 1 — Pathology results from systematic biopsy and targeted biopsy for prostate cancer. (DOCX 16 kb) [file 12894_2017_241_MOESM1_ESM.docx]

Table S1. Pathology results from systematic biopsy and targeted biopsy for prostate cancer.

|  | Totals |  | Biopsy pathology | |  |  | Prostatectomy pathology | |  |
| --- | --- | --- | --- | --- | --- | --- | --- | --- | --- |
|  |  | Insig PCa | | Sig PCa | | Insig PCa | | Sig PCa | |
| Overall PCa | 41 | 15 | | 26 | | 6 | | 20 | |
| SB diagnosed PCa | 27 | 14 | | 13 | | 3 | | 9 | |
| TB diagnosed PCa | 31 | 9 | | 22 | | 2 | | 17 | |

PCa, prostate cancer, SB, systematic biopsy; TB, targeted biopsy.
