# Supplementary figures and images for: Assessment of free-hand transperineal targeted prostate biopsy using multiparametric magnetic resonance imaging-transrectal ultrasound fusion in Chinese men with prior negative biopsy and elevated prostate-specific antigen
Source: BMC Urol. 2017 Jul 5;17:52. doi: 10.1186/s12894-017-0241-3 (PMC5499050; doi:10.1186/s12894-017-0241-3)

Figure S1


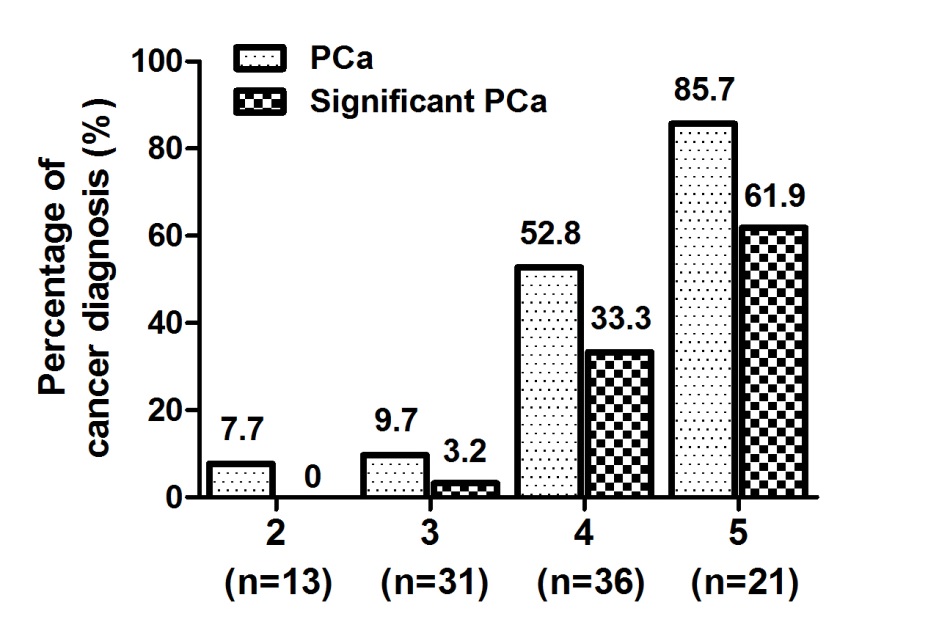

Supplement: Supplementary file 2 — Proportion of all cancers and clinically significant cancers stratified by PI-RADS score according to mpMRI scan. PCa, prostate cancer. (DOCX 138 kb) [file 12894_2017_241_MOESM2_ESM.docx]
